# A note on captive breeding and reproductive parameters of the Chinese pangolin, *Manis pentadactyla* Linnaeus, 1758

**DOI:** 10.3897/zookeys.618.8886

**Published:** 2016-09-19

**Authors:** Fuhua Zhang, Shibao Wu, Cuiyun Zou, Qiaoyun Wang, Shaoshan Li, Ruyong Sun

**Affiliations:** 1 School of Life Science, South China Normal University, Guangzhou 510631, P. R. China

**Keywords:** Age of sexual maturity, birth record, breeding season, gestation period, parturition, Pholidota

## Abstract

The Chinese pangolin (*Manis pentadactyla* Linnaeus, 1758) is a critically endangered species, and documents on its captive breeding and reproductive parameters are scarce. MP8, kept in the Pangolin Research Base for Artificial Rescue and Conservation Breeding of South China Normal University (the PRB-SCNU), gave birth to a male offspring (MP86) on 19 October 2011. The baby pangolin was well developed, with a weight of 120 g and a total length of 23.2 cm. The gestation length of MP8 was estimated to be from 182 to 225d. Reproductive parameters of the Chinese pangolin are discussed based on collected data about this species. The Chinese pangolin has an obvious reproductive seasonality and its gestation length is typically six to seven months. In this observation, estrus and mating principally occurred in a one-year period from February to July. Parturition principally took place from September to February of the next year. Chinese pangolins usually give birth to one offspring at a time (n = 27). Sex ratio at birth was 0.71:1 (♀:♂, n = 12). Average weight for the reproducible females was 3.57 ± 1.38 kg (2.14–6.8 kg, n = 15). We estimated that Chinese pangolins could reach sexual maturity before they were one year old.

## Introduction

The Chinese pangolin (*Manis pentadactyla* Linnaeus, 1758) belonging to the order Pholidota of Mammalia is one of eight extant species of pangolins around the world (Gaubert and Antunes 2005, Wu et al. 2004a), and mainly distributes in the southern area of the Yangtze River, China (Wu et al. 2005). Due to its high value for medicine and food, a high proportion of the population has been illegally hunted and traded. Moreover, its habitats has also been heavily destroyed, thus the population has declined sharply in recent years (Wu et al. 2004b). In 2014, the Chinese pangolin was assessed as critically endangered by IUCN (Challender et al. 2014). Keeping critically endangered species in artificial facilities is *ex-situ* conservation – a temporary measure for urgently saving these species from wild. Chinese pangolins were first maintained in captivity in 1877; and afterwards, at least twenty-four zoos and five universities and institutions tried to keep them. In these practices, however, most of the captive pangolin individuals died within one year (Chao et al. 1993, Cheng et al. 2000, Chin et al. 2011, Clark et al. 2008, Gu et al. 1983, Heath and Vanderlip 1988, Hoyt 1987, Masui 1967, Shi and Wang 1985, Wang 2000, Wilson 1994, Wu 1998, Yang et al. 2001, 2007). Even fewer reproductive records about the female Chinese pangolins’ rutting, mating, pregnancy rate, and births in captivity have been reported. So far, only four cases, three in Taipei Zoo and one at the Research Institute of Forestry of Gaoan County in Jiangxi Province, China, have been made available for study (Chin et al. 2011, Shi and Wang 1985, Yang et al. 2007).

Reproductive parameters, such as age of sexual maturity, breeding season, gestation period, litter size, and sex ratio at birth are basic data for the scientific management of wildlife populations and the prediction of future trends. They are also fundamental for making plans for the captive breeding of pangolins. However, reproductive parameters of the Chinese pangolin are fragmentary, and presented in few studies; data have been mainly based on talking with hunters, dissecting dead pregnant pangolins, and noting rescued pregnant pangolins that gave birth in captivity. Additionally, most of these parameters have been presented as a range, which is not accurate (Chao et al. 1993, Cheng et al. 2000, Heath and Vanderlip 1988, Liu and Xu 1981, Luo et al. 1993, Masui 1967, Ogilvie and Bridgwater 1967, Wang 1990, Wu 1998, Zhu-Ge and Huang 1989). Data from direct observations in captivity are unusually scarce (Chin et al. 2011, Shi and Wang 1985, Yang et al. 2001, 2007). Length of the gestation period of the Chinese pangolin in the Taipei Zoo has varied considerably. Yang et al. (2007) suggested the gestation period was less than 169d, however, Chin et al. (2011) thought that it might be from 318 to 372d. Further research on reproductive parameters of the Chinese pangolin is needed, with more direct observations taken.

In June, 2010, the Pangolin Research Base for Artificial Rescue and Conservation Breeding of South China Normal University (the PRB-SCNU) was built in the village of Sima, in the town of Changping, in the city of Dongguan. There, studies on rescuing and keeping pangolins in captivity were conducted. From then until now, one Chinese pangolin and eight Sunda pangolins (*Manis javanica* Desmarest, 1822) have conceived and given birth to offspring in captivity (Zhang et al. 2015). The information on captive breeding of the Chinese pangolin reported in this paper is intended to enrich the reproductive knowledge and the direct observations of the reproductive parameters of this species. By combining data in this note with existing reproductive data about the Chinese pangolin, the reproductive parameters of this species was discussed in the present paper, findings in this study will then provide guidance for selecting individuals to be mated, determining the season for mating, predicting the parturition time, and creating breeding plans. Data are intended to provide benefits to the management of captive Chinese pangolins, and provide further information for enhancing management practices and predicting population trends of the wild population.

## Materials and methods

### Subjects

Subjects of this study were two wild-born Chinese pangolins marked as MP1 (♂) and MP8 (♀) who were sent to the PRB-SCNU on 24 June and 16 July 2010, respectively. When they arrived, their weights were measured (2.5 kg for MP1 and 3.3 kg for MP8). They were individually housed. Pangolins received treatment for all apparent parasites and disease, adjusted to domestic feeding habits, and adapted to the captive environment.

### Housing

Housing details for the two Chinese pangolins were the same as housing the Sunda pangolin at PRB-SCNU that have been described by Zhang et al. (2015).

### Housing together and mating

The female MP8 and the male MP1 were housed together during the period from 8 March to 20 April 2011. After that, MP8 was housed individually until she gave birth to a baby pangolin. After their separation, we observed the breasts of MP8 to be enlarged, with a little secretion on her papilla. Accordingly, we suspected that MP8 was pregnant, which was proved true several months later by a baby pangolin birth. Undoubtedly, MP8 mated with MP1 during the period in which they were housed together. Unfortunately, we missed observing their mating behavior so we cannot determine the exact mating date.

## Results

### Gestation length and number of offspring

The keeper found a dead pangolin baby (MP86) whose umbilical cord (with a length of 9.2 cm) was still connected to the placenta. The baby was buried in the sandy soil substrates of the nest and under a brick. Examining MP8, we observed breast swelling, indicating her breasts were full of milk. In addition, her vulvae displayed redness with some red viscous secretion. She was sensitive to sound, and in response to noise, became quite alert. When we got close to her, she made a sound of “fu~fu~” and curled herself tightly. Undoubtedly, she was showing strong epimeletic behavior after parturition. When the keeper checked her nest at about 0 o’clock on 19 October, he did not observe MP8 showing any sign of approaching parturition. So MP86 was likely born between 0:00 and 8:00 am of 19 October. Considering MP8 and MP1 were housed together between 8 March and 20 April, the gestation length of MP8 was estimated to be from 182 to 225d. The number of offspring was one.

When the keeper checked the nest of MP8 on the morning of 19 October at approximately 8:00 am, he also found that her bowls for food and water had changed position and had fallen over. Approximately 30 g of artificial food remained in her food bowl. The sandy soil substrate of her enclosure was freshly turned, so we suspected that around the time of delivery MP8 showed several abnormal behaviors with dysphoric emotion.

### Changes of morphology and weight for MP8

**Figure 1.**
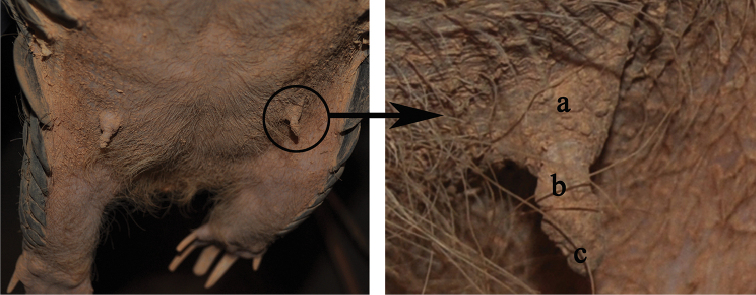
Breast and waxy secretion covering the nipple surface of the female pangolin MP8 for the parturition the day before (by Fuhua Zhang, 18 Oct 2011). **a** breast **b** nipple **c** waxy secretion covering the nipple surface.

**Figure 2.**
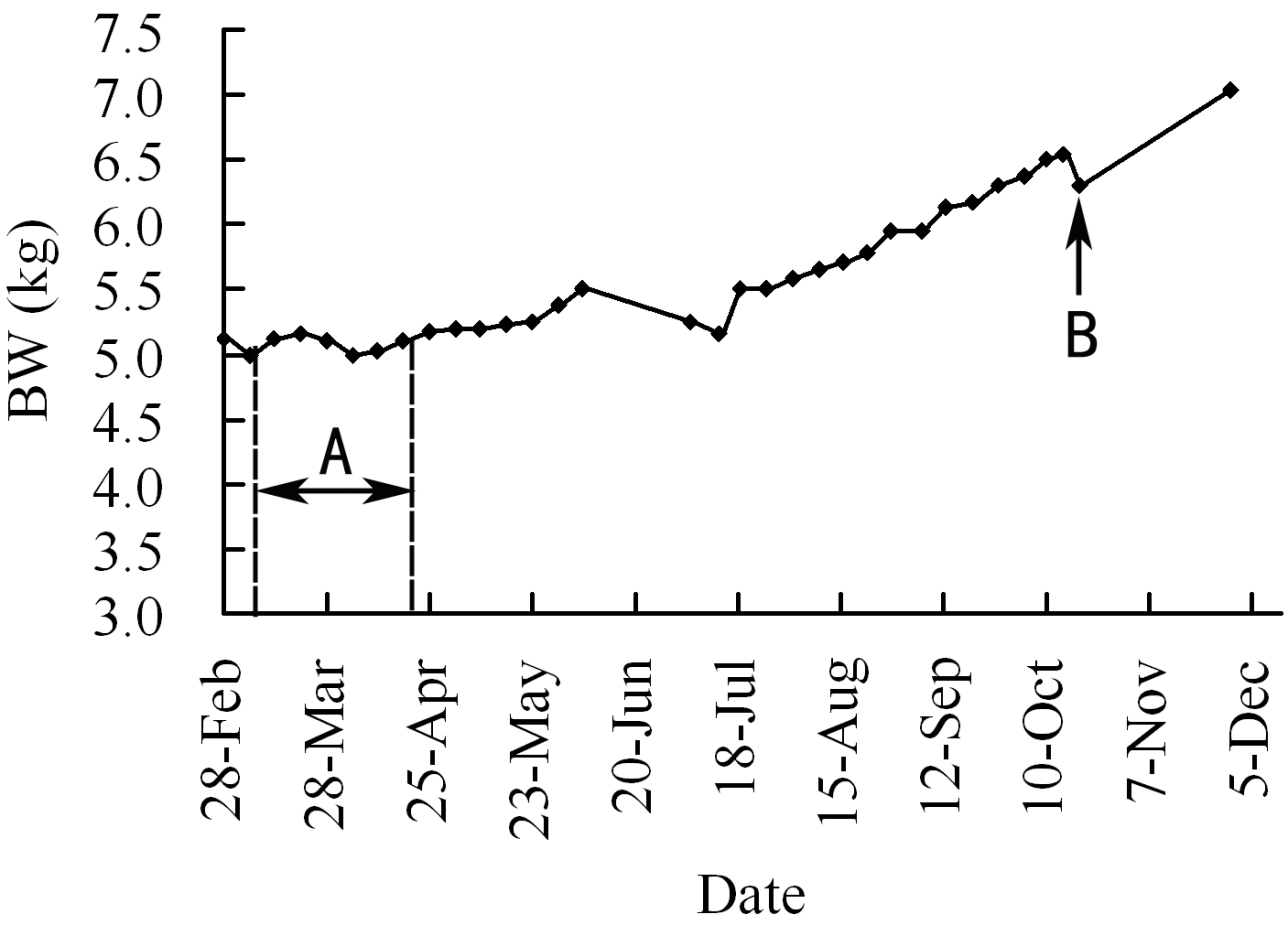
Body weight change of the female pangolin MP8 during the gestation period (from 8 Mar to 19 Oct 2011). **A** duration for MP8 housing with MP1 together **B** date of MP8 giving birth.

During the pregnancy, MP8 did not show any significant morphological changes except for enlarged breasts and concurrent weight gain. Before the pangolins were housed together, her two breasts were only small bumps with a height less than 0.3 cm, with a diameter at the base of the breast of 0.5 cm. Her nipples were not obvious either. After being housed together, the female’s breasts were first observed to be swollen on 24 April 2011; the nipples began to distend outward with some waxy secretion on their surfaces. Her breasts and nipples enlarged gradually, with the breasts becoming significantly fuller and more upright. At the same time, secretions also became thicker. Before her parturition, her breasts had a height of 1.5 cm with a diameter at the base of the breast of 1.1 cm (Fig. 1). Swollen breasts and waxy secretions have also been noted in other reports (Heath and Vanderlip 1988, Yang et al. 2001, 2007). During the gestation period, MP8’s daily intake for food increased from 50 g to 70 g and her weight increased from 5.0 to 6.55 kg – an increase of 31% (Fig. 2).

### Morphological features of MP86

After clearing the sand on its surface, the skin of the head and abdomen of MP86 were found to be broken. It was a male pangolin with its head, limbs, claws, and tail well developed.

**Figure 3.**
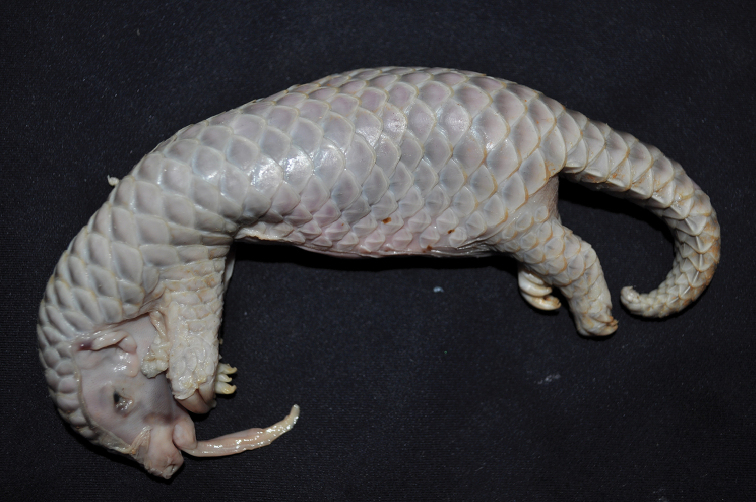
The newborn Chinese pangolin baby MP86 (by Fuhua Zhang, 19 Oct 2011).

Its extraoral tongue extended 2.6 cm. Overlapping scales covered its body, with most closely tied to its surface. Scales tied to the surface were soft and not cuticularized; free parts of the scales were narrow, with a length of about 1 to 2 mm. The scales were grey, and at the base much darker; free parts were milky white, transparent, and membranous (Fig. 3). Delicate and milky white hair was found among some scales.

Its claws were bent and sharp. Their ends were wrapped in soft ivory skin membrane. This membrane structure may be related to preventing the baby’s claws from scratching the dam’s vagina during its transit through the birth canal. Its abdomen was naked, without any scales. Morphological index data are presented in Table 1. According to the degree of MP86’s development and Table 1, we concluded it had been mature and reached full term. MP86 was the offspring of natural childbirth.

**Table 1.** Measurements of the morphological indexes for the baby MP86.

Items	Outcome
Body mass	120 g
Length of head and body	15.6 cm
Tail length	7.6 cm
Total length	23.2 cm
Head length	4.6 cm
Length of the middle claw of fore limb	1.7 cm
Length of the middle claw of hind limb	0.8 cm
Ear length	0.6 cm
Number of rows of scales around middle of the body	15
Number of rows of ridge scales on one side of the body	4
Number of the scales on the edge of one side of the tail	16
Exposed tongue length	2.6 cm

## Discussion

Data from a total of twenty Chinese pangolin births were collected, with five from captive breeding (Table 2). MP86, reported in this paper, was the fifth Chinese pangolin baby in the world to be bred in captivity (Table 2). The Chinese pangolin remains critically endangered, and it is difficult to get samples in the wild. Data from captive breeding records and reproductive parameters of the species in this study are valuable, as information collected enriches our understanding of the reproductive biology and ecology of this critically endangered species.

**Table 2.** The parturition records and estimated gestation period of Chinese pangolins.

No.	Mothers							Litter size	New born babies				Source
	ID	Arrival Data (BW, g)	Mating Date (BW, g)	Mating sites	Giving birth date (BW, g)	Giving birth sites	Gestation length (d)		Gender	BW (g)	TL (cm)	Condition	
1	3#	24 Mar 1984 (3850)	1–30 Jun 1984 (?)	Captive	3 Jan 1985 (?)	Gaoan Forestry Institute	187–216	1	?	165	?	?	Shi and Wang 1985
2	1#	4 Nov 1984 (2350)	Bef 4 Nov 1984 (?)	Wild	5 Feb 1985 (3000)	University of California	>93	1	F	92	21	Viable	Heath and Vanderlip 1988
3	‡	6 Nov 1995^†^ (3855)	Bef 6 Nov 1995 (?)	Wild	15 Feb 1996 (?)	Xiashan Rare Animals Farm	>101	1	M	75	18.5	Stillborn	Wu 1998
4	‡	? (?)	? (?)	Wild	12 Feb 1993 (?)	Taipei Zoo	?	1	?	?	?	Stillborn	Yang et al. 2001
5	‡	21 Dec 1991^†^ (4000)	Bef 21 Dec 1991 (?)	Wild	26 Feb 1992 (?)	Taiwan Forestry Institute	>68	1	M	70	19.2	Stillborn	Chao et al. 1993
6	‡	? 1966 (?)	? (?)	Wild	? Aug 1966 (?)	Rochester Zoo	?	1	?	?	?	Viable	Ogilvie and Bridgwater 1967
7	B	7 Nov 2005 (2800)	Bef 7 Nov 2005 (?)	Wild	20 Sep 2006 (1850)	Taipei Zoo	>318	1	F	52	?	Stillborn	Chin et al. 2011
8	‡	? Sep 2015^†^	? (?)	Wild	23 Sep 2015	Hunter’s home	?	1	F	110	24.5	Viable	Interview hunter
9	‡	? (?)	? (?)	Wild	? Oct ? (?)	Wild	?	1	?	?	?	Viable	Liu and Xu 1981
10	C	2 Oct 2006 (2140)	Bef 2 Oct 2006 (?)	Wild	9 Oct 2007 (4780)	Taipei Zoo	>372	1	M	80	?	Viable	Chin et al. 2011
11	MP8	16 Jul 2010 (3300)	8 Mar – 20 Apr 2011 (5000)	Captive	19 Oct 2011 (6300)	PRB-SCNU	182–225	1	M	120	23.2	?	this study
12	‡	? (?)	? (?)	Wild	1 Nov 2010 (?)	PRB-SCNU	?	1	F	180	26.5	Viable	PRB-SCNU
13	A	9 Jul 2005 (2280)	23–27 Dec 2005 (3600)	Captive	9 Nov 2006 (6050)	Taipei Zoo	317–321	1	F	110	?	Viable	Chin et al. 2011
14	3#	4 Nov 1984 (2950)	Bef 4 Nov 1984 (?)	Wild	14 Nov 1984 (3000)	University of California	>10	1	M	93	20	Viable	Heath and Vanderlip 1988
15	‡	29 Mar 1998 (3000)	15 Jun - ? 1998 (3100)	Captive	28 Nov 1998 (?)	Taipei Zoo	≤166	1	?	?	?	Viable	Yang et al. 2001
16	‡	? (?)	? (?)	Wild	? Dec 1978 (?)	Wild	?	1	?	100	?	Viable	Liu and Xu 1981
17	‡	12 May 1996 (?)	? (?)	Captive	11 Dec 1997 (?)	Taipei Zoo	?	1	M	?	?	Viable	Yang et al. 2001
18	‡	9 Dec 1965 (?)	Bef 9 Dec 1965 (?)	Wild	25 Dec 1965 (?)	Ueno Zoo	>16	1	M	?	?	Viable	Masui 1967
19	‡	? Nov 2014^†^ (?)	? (?)	Wild	? Nov 2014 (?)	Wild	?	1	?	?	?	Viable	Interview hunter
20	‡	? (?)	? (?)	?	1986 or 1987 (?)	Taipei Zoo	?	1	?	?	?	?	Hoyt 1987

† = wild-caught date; ? = Unknown; ‡ = No ID Number; Bef = Before ; BW = Body weight ; TL = Total length .

### Weight change of the pregnant pangolins and survival of the newborns

During pregnancy, the weight of MP8 increased by 31% (1.55 kg) (Fig. 2). It has been suggested that weight increase in pregnant pangolins before delivery would improve the survival rate of newborn infants (Bagatto et al. 2000, Dehnhard et al. 2006, Heath 1987, Heath and Hammel 1986). Chin et al. (2011) reported that the weight of two young pregnant Chinese pangolins increased by 63.89% (from 3.6 kg to 6.05 kg) and 134.0% (from 2.14 kg to 4.78 kg), respectively, before they gave birth to babies. At birth, these babies weighed 80 g and 110 g, respectively, and this modest increase in weight indicates good nutritional status of the mothers, and is helpful for the development of fetuses and births of healthy cubs. Weight gain also contributes to meeting the nutritional need of postpartum lactation so the fetus can survive more easily, unlike a decrease in weight, which would restrict the survival of the cubs or even cause stillbirth. A female Chinese pangolin whose weight decreased by 950 g (from 2.8 kg to 1.85 kg) gave birth to a cub with a weight of 52 g, who died within a few minutes after its birth (Chin et al. 2011).

### Development and health of the newborn pangolins

Twenty birth records of Chinese pangolins have been counted in this paper (see Table 2). Descriptions of the babies were similar. The average weight of baby pangolins was 103.92 ± 37.40 g (52–180 g, n = 12); Total length was 21.86 ± 2.98 cm (18.5–26.5 cm, n = 7). The weight of newborn baby pangolins has been suggested as an assessment criterion for the successful reproduction of Chinese pangolins, as cubs with higher weights usually survive easily (Chin et al. 2011). Among the thirteen viable cubs in Table 2, seven had known weights (No. 2, No. 8, No. 10, No. 12, No. 13, No. 14, No. 16) and were relatively big, with weights over 80 g (the largest was 180 g). Four were stillborn without any vital signs. Three of these had known weights (from 52 to 75 g). They were relatively small and weakly developed. To further describe the degree of development in the newborn Chinese pangolins, we tried to use DI, an index of fatter and thinner, which represents a ratio of the fetus’ weight to its total length. The DIs of the four surviving baby pangolins (Table 2: No. 2, No. 8, No. 12, No. 14) were 4.38, 4.49, 4.65, and 6.79, all of which exceeded 4.2. The DIs of the two dead animals (Table 2: No. 3, No. 5) were 3.65 and 4.05, so below 4.2. We suggest a weight of 80 g and a DI of 4.2 could be used as the scale to describe the degree of development in newborn Chinese pangolins. Of course, more samples are needed to confirm this idea.

### Gestation length

The gestation length of the Chinese pangolin was calculated based on direct observations and published data (Table 2). It seemed that gestation length is not very clear and more data is needed, especially for those collected in captivity. In the present study, the gestation period was from 182 to 225d, in agreement with findings reported by Shi and Wang (1985) (187–216d). Yang et al. (2007) reported a gestation length of less than 169d, and Wang (1990) argued for a period of about eight months (240 d). However, Chin et al. (2011) also reported the gestation length of three Chinese pangolins (A, B, and C) to be 317–321d, > 318 d, and >372 d, respectively (Table 2), all of which were over 300d, the results have large differences from that of the others. The gestation lengths of the Indian pangolin (*Manis crassicaudata* Gray, 1827), Sunda pangolin, and Cape pangolin (*Manis temminckii* Smuts, 1832) were about 165d, 180d, and 139d, respectively (Panda et al. 2010, Zhang et al. 2015, Van Ee 1966), all of which were less than 300d. Wang et al. (1984) reported the gestation length of ninety-seven species of mammals which belonging to thirteen orders. Only a few large-sized species had gestation periods of over 300d, for example, Asian elephants (*Elephas maximas*), Asian rhinoceros (*Rhinoceros unicornis*), and bactrian camel (*Camelus bactrianus*). Small animals tend to have shorter gestation lengths. Perhaps Chin et al. (2011) overestimated the gestation of the three Chinese pangolins (A, B, C).

The gestation period is usually stable as it is genetically controlled (Liu and Zheng 1997). But the gestation period of a few mammals may show larger fluctuations, such as that of the giant panda, whose gestation lengths have been noted as 140.3 ± 20.5d (from 89 to 186d)) (Zhang and Wei 2006). Environmental conditions, health and nutritional status of the mother, delayed implantation, and reproductive hormone levels may each affect animals’ pregnancy lengths (Silk 1986, Yang 2010). The three Chinese pangolins reported by Chin et al. (2011) were kept in very narrow space (1.2×1.2×0.8 m). Thus, the relatively long gestation length of the three Chinese pangolins (A, B, and C) may be related to the disturbance of hormones caused by environmental stress.

### Mating and parturition season

It is usually suggested that the Chinese pangolin has a specific mating and parturition season. Mating behavior has been mainly observed to occur in summer and seldom occurs in April and May or early autumn; births have mainly taken place in winter (Heath 1992a, Liu and Xu 1981, Luo et al. 1993, Wang 1990, Wu 1998, Zhu-Ge and Huang 1989). However, according to the descriptions of thirty-one interviewed hunters, Chao et al. (1993) stated the Chinese pangolins mainly give birth to infants in the spring, between March and May, with the parturition season also later.

In this study, a total of three female Chinese pangolins’ mating times were collected (No. 1, No. 11, and No. 13 in Table 2). Mating for No. 11 and No. 1 occurred between March and May and in June, respectively. Additional mating times for two pregnant pangolins (No. 2 and No. 3) were also estimated according to the times of their death and the degrees of development of embryos in the uterus. Their mating seemed to occur between February and March and between June and July, respectively (Table 3). These few instances appear to show the mating season of the Chinese pangolins mainly occurred between February and July.

**Table 3.** Records of dissection and estimated mating time of the dead pregnant Chinese pangolins.

No.	Mother ID	Arrival date	Estimated mating time	Mating site	Died date	Litter size	Embryo/fetus mass (g)	Fetus gender	Source
1	†	?	?	Wild	? Jan 1982	1	43.0	?	Wang 1990
2	FS3	4 Mar 2011	Feb or Mar 2011	Wild	10 Mar 2011	1	6.5	?	PRB-SCNU
3	MP7	16 Jul 2010	Jun or Jul 2010	Wild	22 Jul 2010	1	1.4	?	PRB-SCNU
4	†	?	?	Wild	? Sep 1978	1	75.0	?	Liu and Xu 1981
5	†	?	?	Wild	? Oct 1979	1	80.0	?	Liu and Xu 1981
6	†	10 Nov 2013	?	Wild	15 Nov 2013	1	46.8	?	PRB-SCNU
7	96004	26 Oct 1996	?	Wild	15 Nov 1996	1	108.0	F	Cheng et al. 2000

† = No ID Number; ? = Unknown

Among the twenty birth records of Chinese pangolins gathered in this study, nineteen of their birth months were known, usually occurring from October to February of the next year, i.e., in autumn and winter (eighteen cases, accounting for a percentage of 94.7%) with a few occurring in August (Table 2). This finding suggests the breeding season of Chinese pangolin is similar to that of the Cape pangolin, but different from the Sunda and Indian pangolins. Regarding the Cape pangolin, mating occurred from late summer to early autumn (between March and May in the southern hemisphere), with the birthing season in winter (from June to September in the southern hemisphere) (Heath 1992b); for Sunda and Indian pangolins, births have also been observed throughout the year (Heath 1995, Mohapatra and Panda 2014, Zhang et al. 2015). Given that direct observations of the mating and parturition for the Chinese pangolin were few, more direct observations and documentation are necessary.

### Litter size and sex ratio at birth

It is commonly suggested that the Chinese pangolin give birth to one offspring at a time. In the current study, MP8 was observed to give birth to a single offspring, coinciding with other breeding records collected (n = 19) in the current study. This is in line with findings from our dissection of seven pregnant Chinese pangolins, where only one fetus was found in each uterus (Table 3). This agrees with birth records for other pangolin species – the Sunda pangolin, Indian pangolin, Cape pangolin, and tree pangolin – where usually a single young was produced (Hoyt 1987, Israel et al. 1987, Menzies 1967, Mohapatra and Panda 2014, Van Ee 1966, Zhang et al. 2015). However, for the Chinese pangolin and Indian pangolin, it is also stated that two baby pangolins can be born at the same time (Liu and Xu 1981, Wang 1990, Prater 2005). In August 2015, we visited China’s Xishuangbanna Natural Reserve, located in Yunnan Province, and interviewed several staff. Interviewees Li Xiaokun (on the staff of this nature reserve) and Lv Xinghua (from Ninger county, Yunnan province) told us that in 1979, they dug out an adult female pangolin and four similar-sized babies (approximately 1.0 kg) in a burrow with a depth of 1 meter in the Wulu River Forest Farm in Mengwang Town, Jinghong City, Yunnan Province. This may indicate that the Chinese pangolin could give birth to four babies at a time.

Sex ratio at birth of the Chinese pangolin has not been reported in other literature. A total of twelve newborn cubs whose gender were recorded in this paper (Table 2), including five females and seven males, suggests a sex ratio of 0.71:1 (♀:♂, n = 12). Female individuals were fewer than males, but this might be ascribed to the small sample size. Natural selection may force parents to regulate the sex ratio of their offspring according to parental ability to invest (Trivers and Willard 1973). Dams in good health have higher levels of investment and tend to give birth to more male cubs. A higher percentage of males mean females have more opportunities to select an excellent mate. This benefits the health of a population and prevents their decline and extinction. It must be kept in mind, however, that a greater number of males will consume more resources, thereby affecting the development of the population (Lumley et al. 2015).

### Weight of the pregnant and puerperal pangolins and age at sexual maturity

The age of sexual maturity for the Chinese pangolin remains unclear. Weights of fifteen sexually mature female Chinese pangolins were recorded in the present study (Table 4), however, it is unclear whether those pangolins were primiparous. Their average weight was 3.57 ± 1.39 kg, with a range of 2.14–6.8 kg (n = 15). Eight females, whose weights were between 2–3 kg, gave birth to offspring, accounting for 53.3% of the total. The weight of the smallest mother pangolin was only 2.14 kg. It has been reported that the six-month-old baby Chinese pangolin could attain a weight of 1.2–2.0 kg (Liu and Xu 1981, Yang et al. 2001), or more (2.7 kg) (Masui, 1967). This indicates that the female Chinese pangolin could reproduce when she was approximately six months old. Chin et al. (2011) argue that the Chinese pangolin could breed before the age of 1–1.5 year, which is in agreement with our conclusion. Sunda pangolins with average weights of 3.49 ± 0.90 kg (1.75–5.54 kg, n = 24) were confirmed to be pregnant, and reached sexual maturity at six to seven months old (Zhang et al. 2015).

**Table 4.** Weight of the collected female Chinese pangolins which have the ability to reproduce in this paper.

No.	ID of the female	Body weight (kg)	Source	Note
1	C	2.14	Chin et al. 2011	pregnant
2	†	2.25	Liu and Xu 1981	pregnant
3	P1	2.35	Heath and Hammel 1986	pregnant
4	MP7	2.42	PRB-SCNU	pregnant
5	†	2.5	Liu and Xu 1981	lactation
6	B	2.80	Chin et al. 2011	pregnant
7	P3	2.95	Heath and Hammel 1986	pregnant
8	†	3.0	Yang et al. 2001	lactation
9	A	3.6	Chin et al. 2011	When mating
10	†	3.855	Wu 1998	pregnant
11	†	4.0	Chao et al. 1993	pregnant
12	FS3	4.1	PRB-SCNU	pregnant
13	MP8	5.17	PRB-SCNU	When mating
14	†	5.6	Interview hunter	lactation
15	†	6.8	Yang et al. 2001	pregnant

† = No ID number.

## Conclusions

1. There is an obvious breeding season for the Chinese pangolin: estrus and mating principally occurred from February to July in a one-year period, and parturition principally took place from September to February of the next year. The gestation length is typically six to seven months.
2. Female Chinese pangolins may reach sexual maturity before one year old, even as early as six months old, or when their body weights reach over two kilograms.
3. During the pregnancy, the Chinese pangolin does not show significant morphological changes except for its breast and body weight.
4. The Chinese pangolin usually gives birth to one offspring at a time. The body weight of all the surviving newborn babies was more than 80 g.
